# Failure Investigation of Layered LFT SB1plus Package after Ballistic Tests for Level IIA

**DOI:** 10.3390/polym13172912

**Published:** 2021-08-29

**Authors:** Cătălin Pîrvu, Lorena Deleanu

**Affiliations:** 1 National Institute of Aerospace Research “Elie Carafoli” INCAS, 061126 Bucharest, Romania; 2 Faculty of Engineering, “Dunărea de Jos” University of Galati, 800008 Galati, Romania

**Keywords:** aramid fiber, ballistic test, failure mechanism

## Abstract

The main objective of this study focuses on designing and testing body protection systems using advanced materials based on aramid fibers, for high impact speeds of up to 420 ± 10 m/s. Ballistic applications of aramid fiber-based composites mostly include soft body armors. The investigation of the failure mechanisms identifies issues of protective fabrics, major challenges and technological problems for efficient development of these systems. The authors present an investigation on the failure processes and destructive stages of a ballistic package made of successive layers of LFT SB1plus, a trade name for a multiaxial fabric by Twaron Laminated Fabric Technology (LFT), taking into account the particular test conditions from NIJ Standard-0101.06 Ballistic Resistance of Body Armor. The main parameter of interest was the backface signature (BFS), but also details of projectile arrest and SEM investigation could offer arguments for using this material for individual protection. For the reported tests, the maximum and minimum values for BFS were 12 mm and 24 mm, the mean value being 18.66 mm and the standard deviation being 3.8 mm.

## 1. Introduction

For hundreds of years, metallic materials have been used not only for body armor, but also for the protection of larger objects, such as vehicles, which is called “heavy protection”. However, just a few decades ago, at the end of World War II, lighter solutions emerged, especially for military personnel, in the form of nylon ballistic vests. However, those did not come close to the current level of ballistic protection offered by aramid fibers, yarns and fabrics, included in personal armors. Another advantage of ultra-fine polymer filaments (not only aramid [1]) is that they offer extraordinarily flexible fabrics, which favors a high level of comfort for the wearer.

Ballistic applications of aramid fiber-based composites mostly include soft body armors. The mechanical properties of the aramids and the ballistic effects on its fabrics and composites have been investigated in several studies [2,3,4,5] that involved both experimental testing and FEM (finite element method) [6,7,8,9] and established the effectiveness of the ballistic protecting system and the protection level of bulletproof vests. Recent reviews on ballistic protection [10,11] point out interesting comments on failure mechanisms and very particular solutions in combining different materials for facing very different threats. Research has involved aramid fibers with different architectures, from simple or treated woven fabrics [12] to 3D fabrics [13] and unidirectional or multiaxial non-crimp fabrics, each solution being simulated with particular model conditions and tested for a specific threat, the conclusion of the documentation conducted by the authors being that each solution has to be experimentally investigated and the failure mechanisms understood in order to offer a design with very strong statistical reliability before use in combat.

Bajya et al. [7] recently presented an experimental report on ballistic protection and failure mechanisms of soft armor packages made of different structures, including woven and unidirectional fabrics. Packages made of para-aramid woven fabrics and unidirectional laminates and hybrid ones were developed and subjected to ballistic impact against 9 mm lead core bullets, at an impact velocity of 430 m/s. The ballistic performance of each structure was evaluated in terms of backface signature (BFS), perforation ratio (PR) and the expansion of the bullet. The unidirectional aramid packages failed mainly due to fiber break, including fibrillation, debonding and delamination. The fiber orientation was only (0°/90°) × 2 and the surface density of this type of fabric was 230 g/m^2^, with this inducing enough flexibility to allow for a too high BFS as compared to other solutions offered by these authors.

In general, ballistic threats are either from bullets or fragments. Bullets can be defined as projectiles of different shapes and consistencies, fired from weapons such as pistols, revolvers and rifles. Fragments, on the other hand, may also result from explosions (for example, detonations of a grenade) or from the target the projectiles had already hit.

The various kinetic characteristics and deformation behaviors of such a wide range of bullets and fragments must require a customized study for each of this multitude of different projectiles and for the design required to face an impact process, in order to have an effective ballistic protection.

Even if, nowadays, many models and simulations have been reported, from micro [14,15], meso [13,16], to macro [17,18] scales, the tests remain the ultimate and restrictive step in adopting a certain type of individual armor, generally based on aramid fabrics.

An active field of research concerning the impact resistance of aramid fabrics involves the study of ballistic performance, from destructive investigation based on standard structures of packages [19] or structures involving new entries such as aerogels [20] and methodologies accepted by the interested parties. The standard structure of a ballistic package involves a certain number of layers of material or semi-finished product (as fabrics) for ballistic protection, which can vary within fairly wide limits between 8 and 40 layers (or folds), depending on its architecture and the material characteristics for the ballistic protection.

The aim of this study is to evaluate a particular multiaxial fabric, LSF SB1plus, produced by Teijin [21], for designing body armor for the threat level IIA as classified in Ballistic Resistance of Personal Body Armor NIJ Standard–0101.04 [22], to identify failure mechanisms and to measure a well-accepted characteristic, backface signature (BFS), for the designed ballistic protection package. The authors measured BFS and investigated failure mechanisms of this particular package in order to evaluate it as a future application in body armor.

## 2. Material and Testing Campaign

Even though fabrics based on aramid fibers are lightweight and offer better protection, the designer will be forced to reduce the number of fabric layers required without compromising the effectiveness of the final protecting armor to reduce the cost. In general, aramid fibers are 43% lighter than glass fibers, as strong as E-Glass, ten times stronger than aluminum and are close in strength to carbon fibers (in tensile tests). They also have good dimensional stability with a slightly negative coefficient of thermal expansion (−2.4 × 10^−6^ 1/°C) and could resist chemicals with the exception of a few strong acids and alkalis. Aramid fibers exhibit excellent stability over a wide range of temperatures for prolonged periods with no strength loss at temperatures as low as −196 °C and do not melt but will start to carbonize at approximately 427 °C [10].

**Figure 1:**
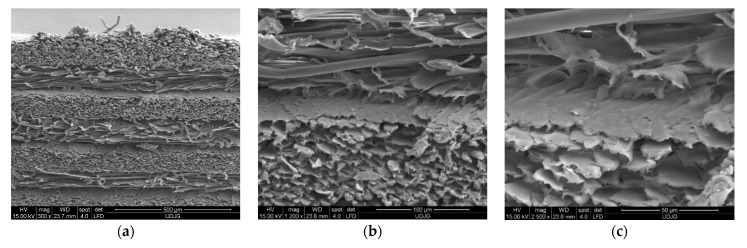
SEM images of the LFT SB1plus layers after being cut by the help of a power guillotine. (**a**) Magnification ×300; (**b**) magnification ×1200; (**c**) magnification ×2500.

The layers are available in the form of fabrics in multidirectional yarns (0°, 90°, −45°, 45°) (Figure 1), namely LTF SB1plus (a trade name for a multiaxial fabric by Twaron Laminated Fabric Technology) (Arnhem production facility, Arnhem, Netherlands), and they are laminated together with a very thin foil of resin [21]. These fabrics can be combined into various structures and composites, each type having unique protection performance, forming ballistic packages with different uses and providing protection for a wide range of threats.

A recent tendency of research in trying to improve the response of aramid fabrics to ballistic impact is 3D architecture [2], but fabrics with unidirectional layers with different angle orientations in sublayers are also intensively used for body armor, due to the fact that the strength of the yarns is not “consumed” in bending them in a 2D or 3D architecture. Of course, technology is obliged to find the adequate film and yarns for maintaining these multiaxial orientations in a single layer.

The architecture of the ballistic package is usually made of the same type of layer or combinations of two or more materials, the designer being interested in comparing ballistic packages with different numbers of layers in their structure [6]. For individual armor, fabrics could be used as delivered, without adding adhesive among layers. No influence of stitching the layers on the ballistic resistance properties of the package has been noticed.

Taking into account the stated criteria, the development of a type of soft armor, from the same aramid textile product, is quite numerous and technologically advantageous.

Designing a new individual protective armor requires adequate layers and yarn architecture, taking into account the particular response of the system to particular threats and the protection degree, including here the limitative request as an acceptable value of backface signature (BFS).

**Figure 2:**
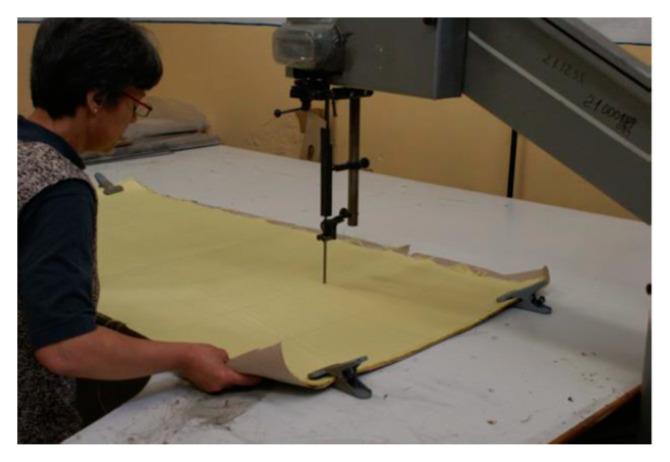
Cutting process of the layers for the ballistic package.

The packages were obtained as successive layers of Twaron LFT SB1plus, as delivered in rolls by the producer, Teijin Aramid [21]. Twaron LFT SB1plus is a fabric of four layers of unidirectional Twaron yarns, with orientations (0°, 90°, +45°, −45°), laminated together with a very small amount of polymer [21]. It has a surface density of 430 g/m^2^ and the theoretical mass of the 12-layer package of 500 mm × 500 mm is 1290 g. The manufacturing of the packages includes the cutting and coupling of the fabrics (in order to have a narrow variation in their weight), sewing and control. The package has the dimensions 500 mm × 500 mm, with an area of 0.25 m^2^, between recommended values by NIJ-C-4 (0.23 m^2^) and NIJ-C-5 (0.3 m^2^) for high and very high surfaces (Figure 2).

After being cut, 12 layers formed a package, this number being selected by the authors after preliminary tests on 8 layers that gave both results as total and partial perforation. In previous works [6,17], macro simulation on equivalent layers gave similar results to the tested package with the same number of layers (bullet was stopped on the last broken layer for a virtual package of 8 layers and simulation on a 12-layer package gave satisfactory results; thus, this number of layers (12) was used in forming a protective package).

The projectile was an FMJ (Full Metal Jacket) 9 mm bullet, as required for protection level IIA as given in NIJ Standard-0101.06/2008 [23]. The layers were laterally fixed by sewing on two sides, on a central location for a length of approx. 90 mm, in order to maintain its integrity and the layers’ order. The packages were tested in a controlled environment enclosure (temperature kept in the range of 20 ± 5 °C and relative humidity of 50…65%, atmospheric pressure of 760 ± 15 mm Hg), using a ballistic smooth barrel, the measured velocity of the projectiles being 420 ± 10 m/s. There were used a chronograph Oehler 43 (Oehler Research, Inc., Austin, Texas, USA) for measuring the impact velocity and a firing table with compensated rebond.

## 3. Ballistic Impact Testing

In practice, in order to assess the impact resistance of a protection system, there are reference standards that propose methods of assessment, such as NIJ Standard-0101.06/2008 [23] and NIJ 0101.04/2004 [22], the results giving the possibility to include the system in a level of protection [10].

**Figure 3:**
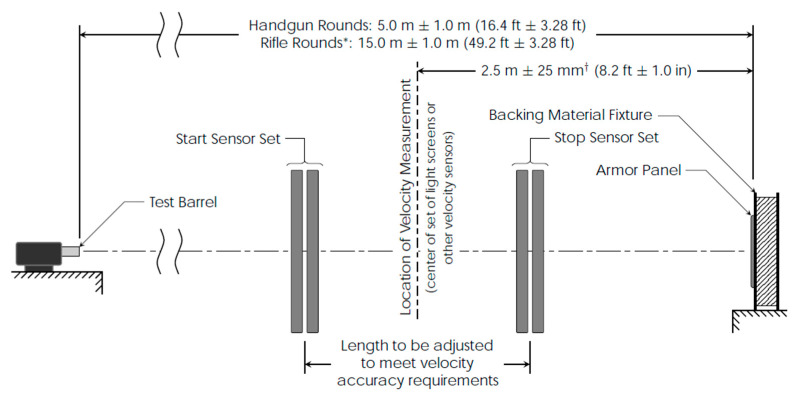
Firing arrangement equipment, Reprinted with permission from ref. [23], Copyright Year Copyright Owner’s Name. * For rife rounds the length may be further adjusted to minimize yaw at impact; however, in such cases the yaw at impact must be experimentally shown to be less than 5° and reasonably close to minimal. ^†^ Tolerance for 0° shots. For 30° and 45° shots the tolerance shall be +25 mm/−190 mm (+1.0 in/−7.5 in).

The assessment of the ballistic resistance of the studied protection packages was performed according to NIJ 0101.04/2004 (Figure 3) [22], by firing a 9 mm caliber bullet, with an initial speed of 420 ± 10 m/s, from a distance of 3 m (normal conditions), with shots fired in a laboratory facility.

The testing of the realized protection packages in order to evaluate their resistance to the 9 mm FMJ bullet was discussed, taking into account the resistance to penetration through the depth of the trace left in the support material (ballistic clay), namely backface signature (BFS). The assessment of the total penetration of a package is in many cases simple, when it is found that there is a hole with a diameter at least equal to the caliber of the bullet and that the entire bullet passes through it.

When testing personal ballistic protection equipment, the trauma on the human body is assessed by the depth of the BFS imprint that is formed in the ballistic clay on which the sample is fixed. The standard admits materials and protection systems as satisfactory when they produce an imprint in the support clay that does not exceed 44 mm [23].

The following steps are performed when testing personal armors or parts of it:

- The test equipment is positioned in the clamping support, at the distance imposed for each piece of equipment from the mouth of the barrel; the types of weapons and ammunition required for levels II and IIA are verified [23];
- The bullet speed measurement system is positioned, starting with the distance of 2 m from the mouth of the barrel, so that the frames of the system are in planes perpendicular to the firing direction; the distance between the frames of the system is 0.5 m and the distances are measured with an accuracy of 1 mm;
- Firing is executed on the test package.

## 4. Results and Discussion of Failure Mechanisms

Factors influencing ballistic protection against a specified threat include those characterizing the target material(s), in terms of fiber and yarn properties, weave architecture, surface density and the number of plies, boundary conditions and those characterizing the impact (impact velocity, impact angle, projectile shape and materials, etc.) [10].

How do we evaluate the ballistic response of body armor? The investigation is done at macro scale and the tests produce clear results: if the target resists, the backface signature could reveal the degree of protection and, if the target does not resist, the residual velocity would indicate how far away the protective solution is from the requested parameters, but in a qualitative way. However, this scale does not offer answers to improve the ballistic resistance, and the study of how projectiles and targets are destroyed at lower scale is of great interest. Thus, researchers will investigate the target and projectile failure mechanisms using optical and scanning electron microscopes, high speed cameras and spectrometers, etc.

**Figure 4:**
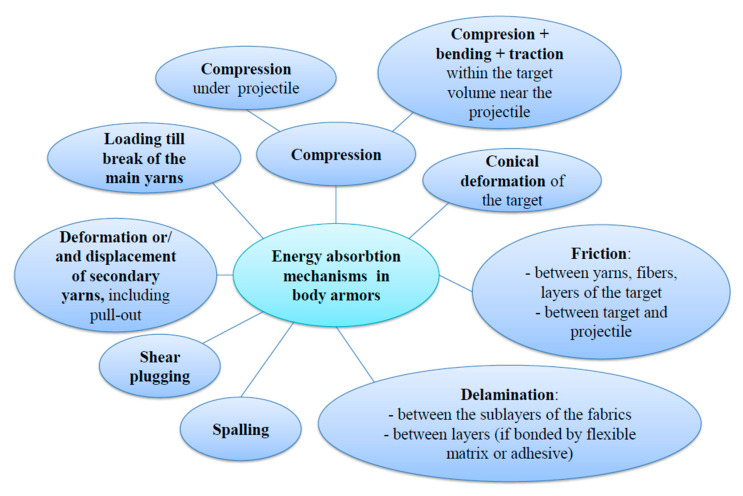
Damage mechanisms for packages based on fabrics.

A synthesis of the damage mechanisms under impact is given in Figure 4.

Based on a general overview [10,24] on how the kinetic energy of the projectile is absorbed, this study aims to identify damage mechanisms for a particular package of 12 layers of LFT SB1plus when impacted with a 9 mm FMJ:

- Compression of the package directly under the bullet;
- Compression in the target volume surrounding the impacted zone;
- Conical deformation on the package backface;
- Loading the main yarns that face the higher strain; these yarns tend to fail when the induced tensile strain of these yarns exceeds the ultimate strain;
- Deformation and/or displacement of secondary yarns;
- Delamination, especially on the layers situated towards the back of the target;
- Shear plugging;
- Spalling of the already broken layers (yarns);
- Friction between the projectile and the target and friction between yarns and layers.

The secondary yarns are close to the main ones, absorbing energy by their strain distribution within the yarns, and the highest values are found near the top face of the deformed cone.

As the friction coefficient is difficult to measure for high impact velocities, simulation offers a fair evaluation by covering a large range of values for friction coefficient. The friction coefficient could have lower values, being more intense when the bullet is forced to be deformed inside the target.

When the bullet hits the target, because of the sudden drop in contact force, only the upper few layers fail by shear as the shear wave propagates along the thickness direction. Then, the undamaged layer absorbs the residual kinetic energy of the projectile through creating a cone-shaped deformation. For woven fabrics, this deformation could be rhomboidal, the rhombus diagonals being almost oriented along the warp and weft yarns. Multiaxial unidirectional fabrics have an intermediate behavior, that is, the shape of deformation could be almost conical.

Shear plugging is one of the major damage mechanisms during impact for energy absorptions by the targets. This process occurs due to the initial impact contact force between the projectile and the target, resulting shear stress through its thickness, around the periphery of the projectile. If shear plugging stress exceeds the ultimate shear plugging strength, the target will fail. The failure by shear plugging is not symmetrical as if the first yarns will be broken by shear, these yarns are de-stressed and able to be displaced when the projectile advances.

During an experimental campaign, the investigation of failure mechanisms points out the ballistic performance of a specific target configuration when hit by different projectiles, with different impact velocities.

**Figure 5:**
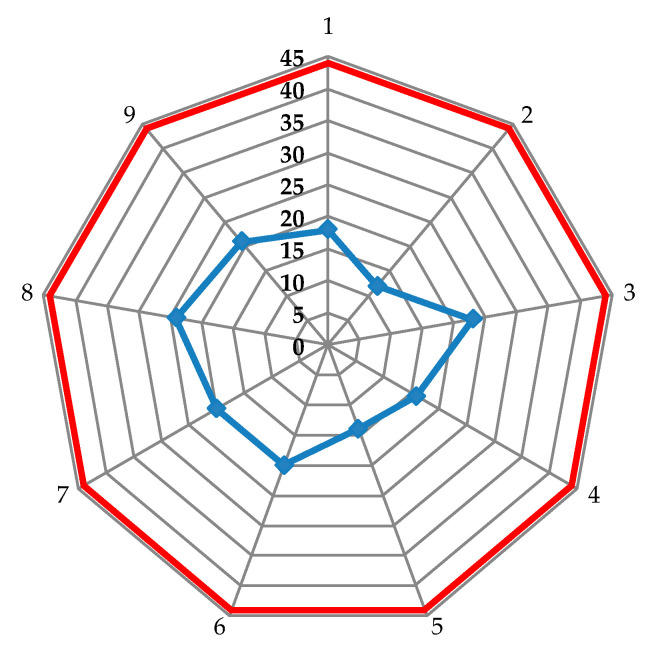
Data for BFS measured for 9 fires on three packages made of 12 layers of SB1plus fabric.

The test results from nine fires on three packages made of 12 layers of SB1plus are plotted in Figure 5, on a web chart, with the red line being the accepted limit of 44 m [23] and the blue points being the values measured as the depth of the imprint in the ballistic clay.

**Figure 6:**
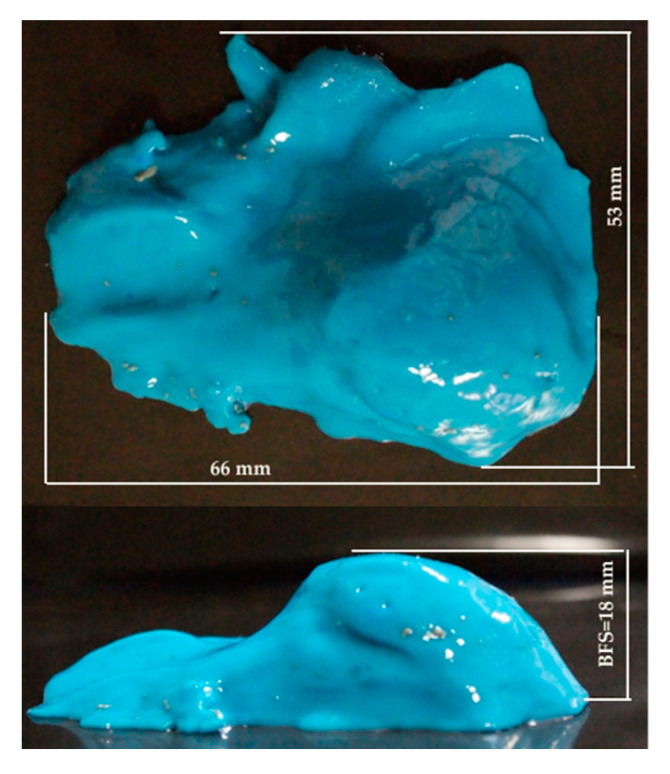
Cavity shape in the ballistic clay, obtained with a molding resin.

Figure 6 presents the shape, as taken with a mold resin, produced by a fire, in the ballistic clay behind the package in order to highlight the importance of this characteristic for body armor. From this study, the authors want to suggest a more detailed evaluation of the entire shape of the backface signature, not only its depth in the ballistic clay, with BFS being of 18 mm here.

An investigation of the destruction of flexible packages was carried out with the help of a professional camera to highlight characteristics dependent on fabrics’ architecture and the number of layers. A detailed, photographic study on each layer of the tested ballistic packages was performed to point out the failure processes in stages of the layers.

**Figure 7:**
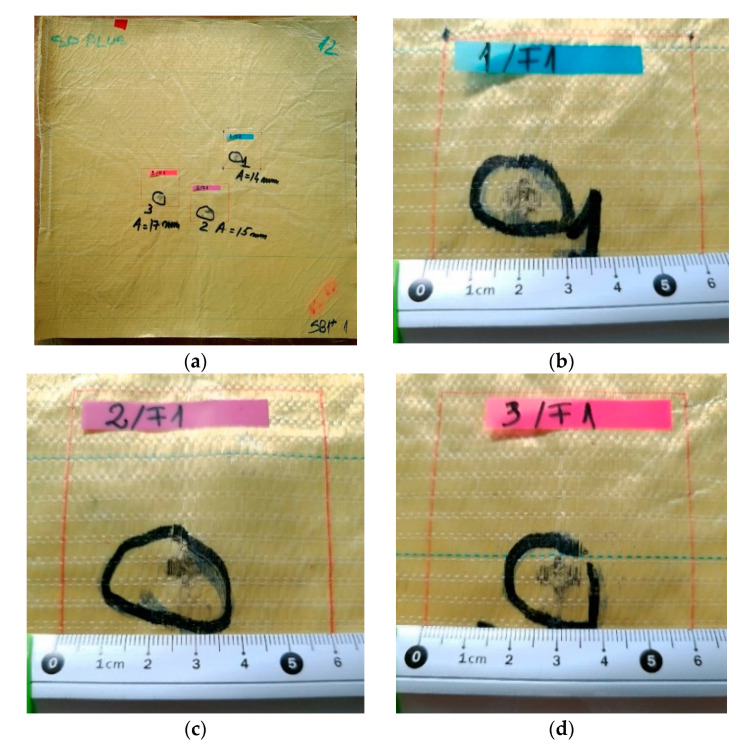
Frontal view (**a**) of a 12-layer LFT SB1plus package: (**a**) layer 1 (face) and details of the fires on the first layer of the package: (**b**) fire 1, (**c**) fire 2 and (**d**) fire 3. BFS was noted as A on the photo.

**Figure 8:**
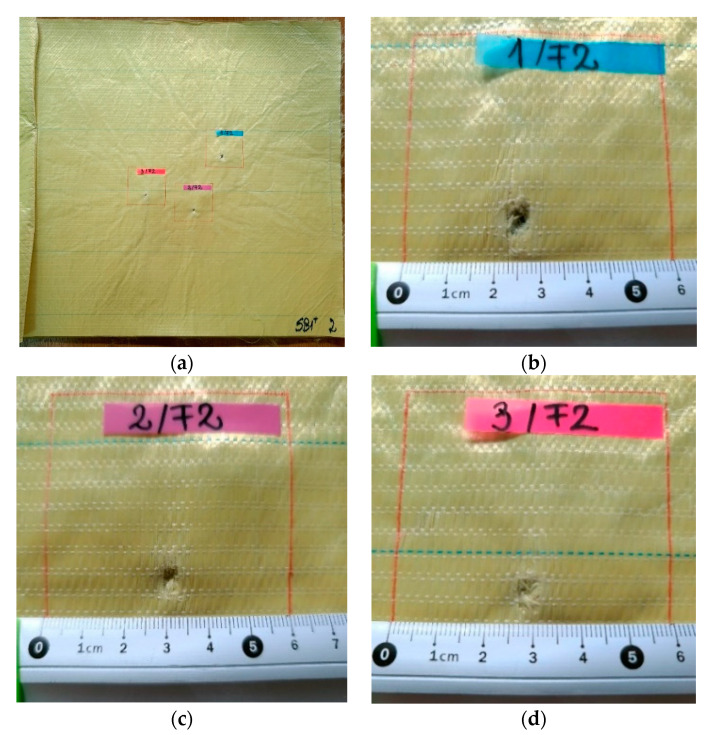
Frontal view of the sample with 12 layers of LFT SB1plus: (**a**) layer 2 (face), with details of the bullet holes of (**b**) fire 1, (**c**) fire 2 and (**d**) fire 3.

**Figure 9:**
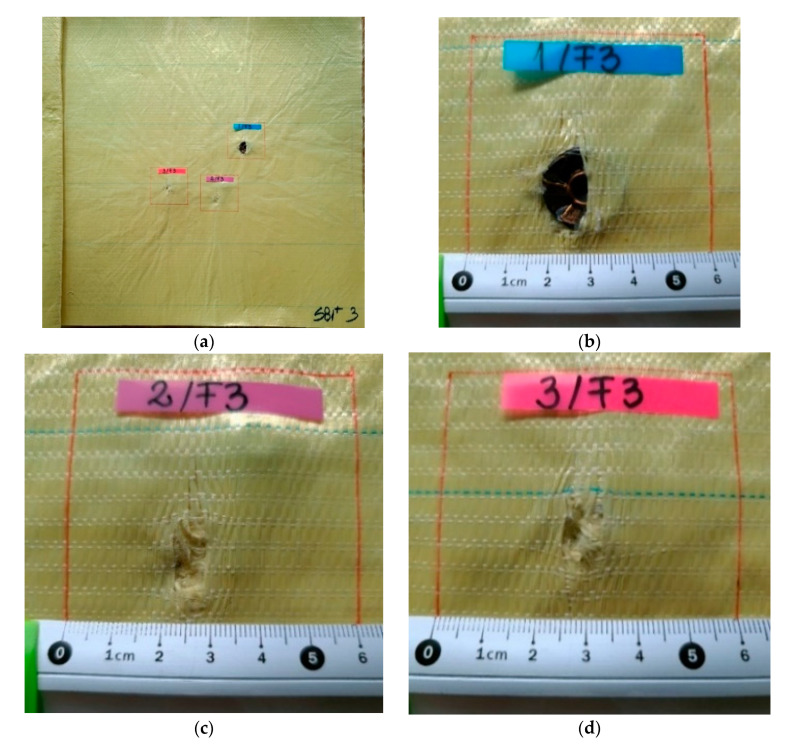
Frontal view of the sample with 12 layers of LFT SB1plus: (**a**) layer 3 (face), with details of the bullet holes of (**b**) fires 1, (**c**) fires 2 and (**d**) fires 3.

**Figure 10:**
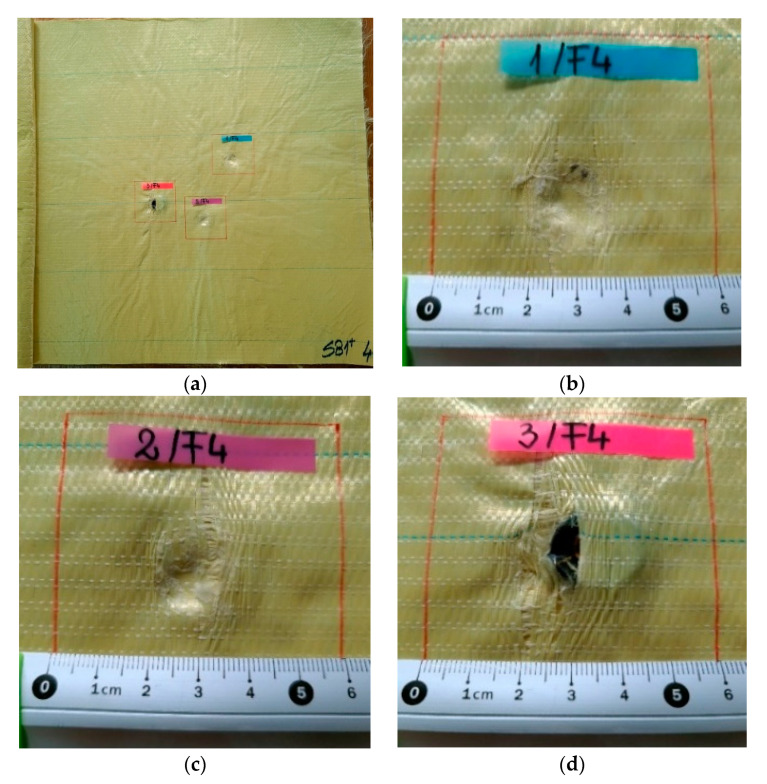
Front view of sample made of LFT SB1plus: (**a**) layer 4 (face), with details of the penetration: (**b**) fire 1, (**c**) fire 2 and (**d**) fire 3.

**Figure 11:**
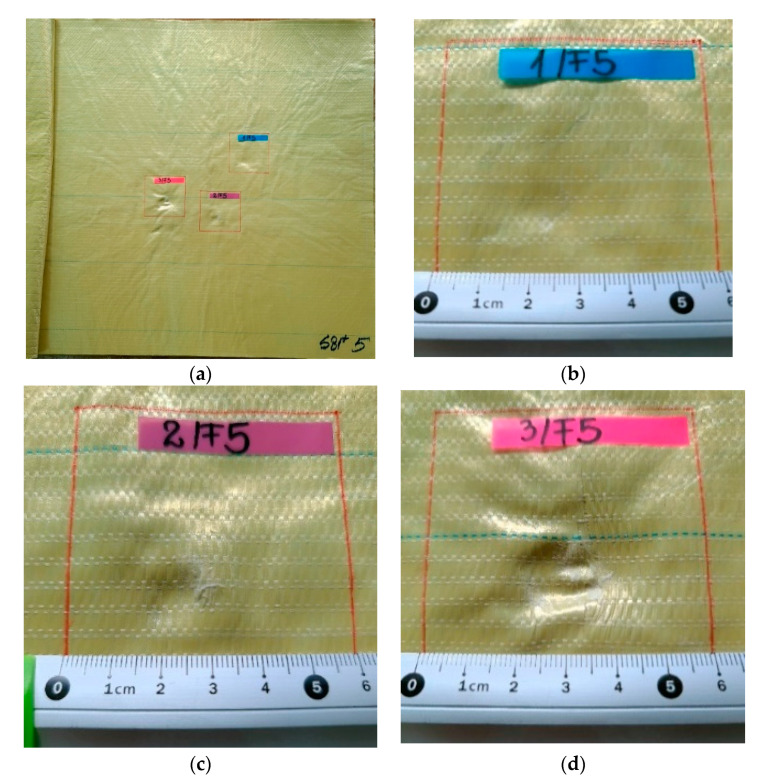
Frontal view of a package made of layers of LFT SB1plus: (**a**) layer 5 (face), with details of (**b**) fire 1, (**c**) fire 2 and (**d**) fire 3.

Figure 7, Figure 8, Figure 9, Figure 10 and Figure 11 show photographs of LFT SB1plus layers from a 12-layer package. It should be noted that the tests were performed under conditions of a small variation for the bullet velocity (420 m/s ± 10 m/s). The angle of impact is normal on the target surface, the deviations being 5% at the mouth of the barrel.

In the details of the photos, for each fire the main yarns (broken under the projectile) and the secondary yarns (those affected by the penetration of the projectile, by twisting, firing and scattering) may be counted.

One may see the total penetration of the first layer in Figure 7: (a) general view from the impact direction and (b), (c) and (d) details of each of the three fires on the same target. The second layer is presented in Figure 8: (a) general view of the designed ballistic package, and the localized ruptures of the yarns is highlighted, for each fire, in (b), (c) and (d). This type of failure occurs when all the fibers of the thread break in almost the same place. The two causes of the breaking of the yarns in layer 1 are their stretching along the length next to the contact with the projectile and the shearing on their thickness, observed in Figure 7 (detail b). Theoretically, the fibers in the yarn will fail when the induced strain exceeds the strain at break, but the process is dynamic (depending on strain rate) and statistic, given the large number of fibers in a yarn.

Starting from layer 3 (Figure 9) and in layer 4 (Figure 10), the enlargement of the holes is observed (due to the lateral plastic deformation of the projectile when it starts to be stopped), and also the process of pulling the threads. The tear-off of the yarn (even partially) from its fabric architecture occurs when the yarns are not broken, sometimes only on one side of the projectile [6].

Layer 4 is the last layer in the SB1plus LFT package through which the bullets passed or stopped. In the current case of failure, the first four layers have the role of slowing down and even arresting and deforming the projectile.

Starting from layer 3 (Figure 9) and in layer 4 (Figure 10), the widening of the holes is observed (due to the lateral deformation of the projectile when it starts to be stopped, and the process of pulling the yarns). The first fired bullet (Figure 9a) was found in the slot produced in the third layer, the second was found between the third layer and the fourth one (see the mark on Figure 10c) and the third bullet fired on the package had a similar position and shape, but advanced one layer (it was arrested between the fourth layer and fifth one). For body armor, it is very important that successive fires do not create failures with very different and progressive damages on the package. The authors consider this response promising in facing multiple and localized fires.

The quality of the material SB1plus is noticed by the high degree of resemblance of the penetration zones and even by the size of zones with broken and torn-off yarns.

Layer 5 (Figure 11) shows traces of crushing (compression), circular in shape, which would be justified by the architecture of the LFT SB1plus semi-finished product (with the orientation of the yarns 0°/90°/45°/−45°), inducing a tendency to uniformity of the properties of the fabrics in four sub-layers and, therefore, of its answer.

Layers 6 and 7, theoretically, have the role of retaining (arresting) the projectile and dumping its tendency to advance together with the fabrics. These layers and the following ones are also responsible for reducing the BFS.

**Figure 12:**
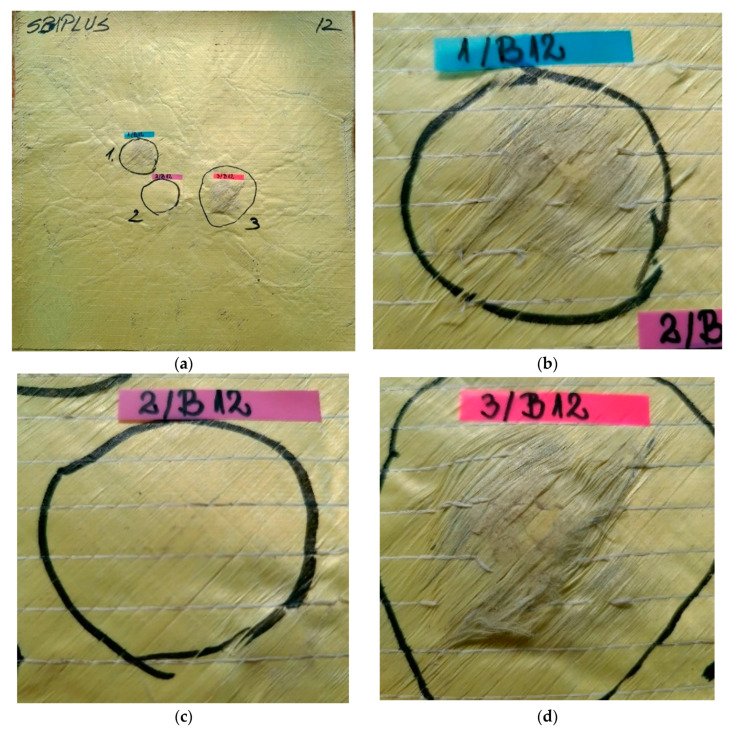
Back view of package made of 12 layers of LFT SB1plus: (**a**) layer 12 (back), with details of (**b**) fire 1, (**c**) fire 2 and (**d**) fire 3.

Figure 12 presents the last layer of the package: (a) the general view being taken from the opposite direction of the impact (named back face in this text), (b) the print on the last layer of fire 1, where aramid fibers are not broken, only the auxiliary (white) yarns that keep the resistant aramid yarns in their aligned position, (c) for fire 2 the yarns are not broken but (d) for the last fire, there are several bundles of fibers broken due to the large deformation on this layer.

After testing, it can be seen that the arching in the orthogonal yarns is more dominant in the back layers of the multilayer package, where the projectile tries to enter through an edge-like approach after being considerably slowed down by the initial layers [8]. The existence of the phenomenon of passing through the fabrics usually produces a hole smaller than the diameter of the projectile, with a smaller number of yarns being broken as compared to the number of yarns that intersect the projectile.

**Figure 13:**
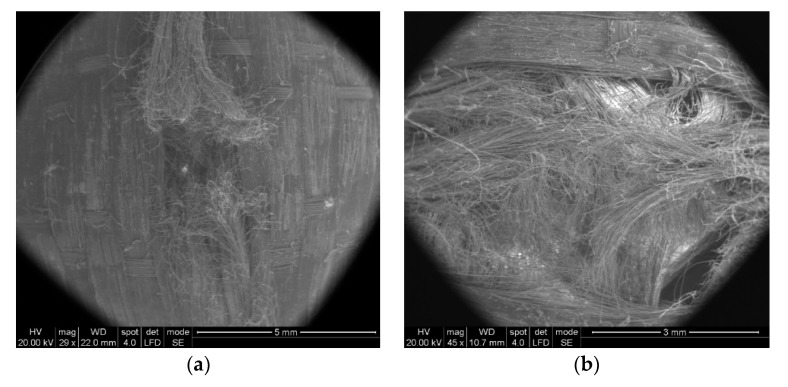
Aspect of broken yarns from the ballistic protection package made of LFT SB1plus: (**a**) face of layer 1 (fire 1); (**b**) face of layer 2 (fire 1).

In Figure 13a, two broken main yarns can be observed on the first layer of unidirectional yarns. The ends of the broken yarns relax after being tensioned, hence the appearance of scattered fibers. Figure 13b shows the deviation of the secondary yarns from layer 2 (from the four substrates of a layer) and the more disordered aspect of the fibers, as compared to layer 1.

**Figure 14:**
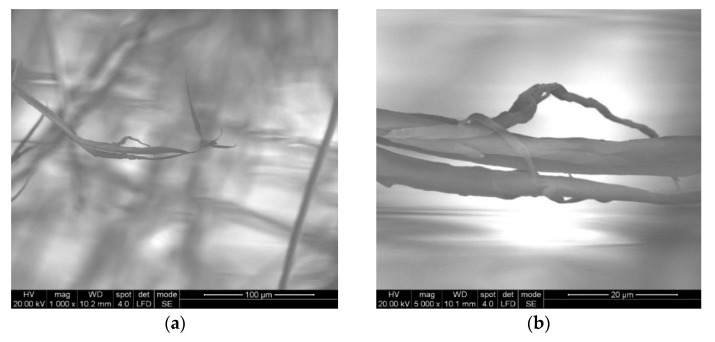
Details of fibers from the ballistic package LFT SB1plus: (**a**) fibril from front layer 2; (**b**) detail from (**a**).

**Figure 15:**
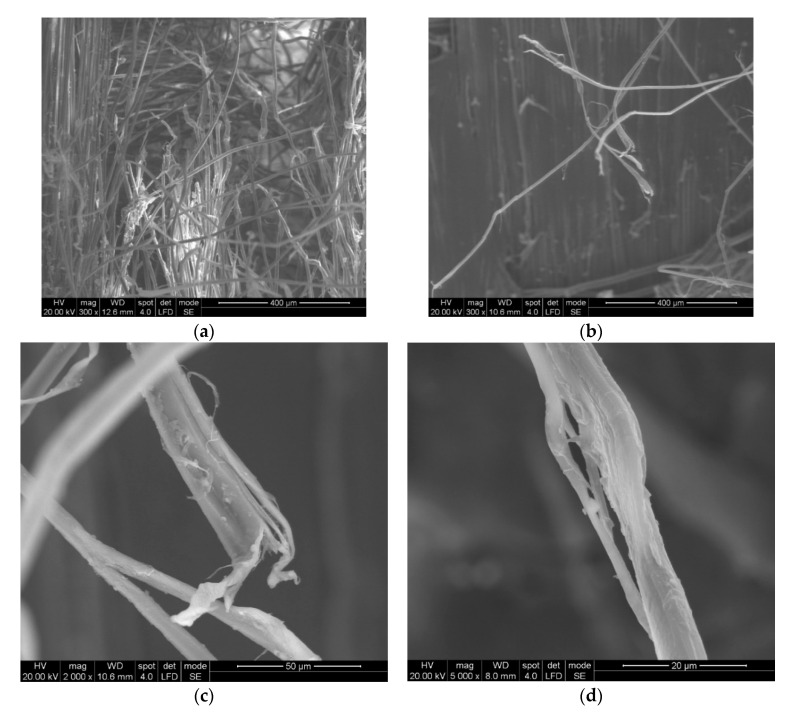
Details of the broken fibrils from the 2nd layer of the LFT SB1plus ballistic package. (**a**) layer 1, front view of the broken fibers, from the impact direction; (**b**) details of layer 1, front view of the broken fibers; (**c**) details of a higher magnification of a fiber; (**d**) details of (**b**) with non-uniform fibrillation and necking.

Some particular aspects of the failure of aramid fibers could be highlighted with the help of SEM images for the tested ballistic package of 12 layers. According to SEM images, breaking a fiber can be performed by fibrillation (Figure 14), shearing (Figure 15), necking (obviously, by tensile stress, as one may see in Figure 15 details (c) and (d) and twisting).

In Figure 14, by the help of scanning electron microscopy, one may see the fibrillation, thinning and rupture of the aramid fibrils, resulting from the process of destroying a thread on the 2nd layer.

The fibrillation at the broken ends of the yarns (Figure 14a,b and Figure 15c) or along the yarn (Figure 15d) is a type of failure favored by the abrasive action of the projectile on the length of the fiber, but it is also dependent on the local traction characteristics of the yarn fibers. Of course, the location of the fibrillation is a defect of the molecular chains or their arrangement in the fiber. In layered systems, friction between layers results in abrasion friction among fibers, inducing local scratches that diminish the fiber resistance. All projectiles that have the ability to penetrate through the fabric also cause the scratching and/or splitting of the fibers, which favors the appearance of fibrillation, a specific failure of aramid fibers.

Figure 15 shows the details of some fibers damaged by fibrillation; there are noticed discontinuities in the fiber and the thinning of the fibrils that supported the load and ruptures of fibrils resulting from tensile and shearing loading (deformed breaks with less deformations, as a cut) during the destruction process.

**Figure 16:**
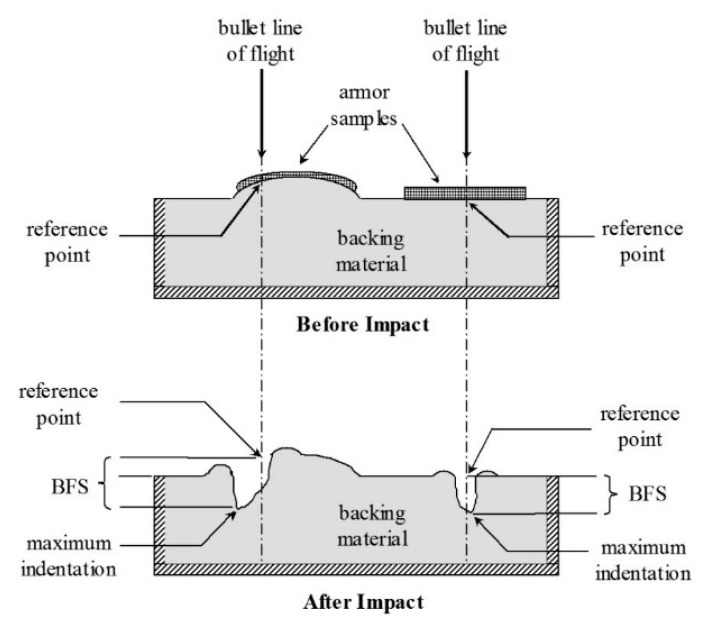
Measuring BFS in agreement with Ballistic Resistance of Body Armor, NIJ Standard-0101.06 [23].

The values for BFS (backface signature) for the LFT SB1plus package are given in Table 1 and are measured according to Ballistic Resistance of Body Armor, NIJ Standard-0101.06 [2] (Figure 16).

Imprints in the clay support material, characterized by the BFS, for a new armor package, were analyzed to determine whether the armor will provide adequate protection against trauma (without perforation/penetration of the package). The requirements of the NIJ [23] state that all depths of the measured traces in the support material obtained from fires falling within the firing requirements must not exceed 44 mm, or if the BFS exceeds 44 mm then there must be a coefficient of 95% confidence that 80% of the depths of the traces in the support material will be 44 mm or less. Under no circumstances should a trace in the support material exceed 50 mm.

Measured BFSs for a new armor package were analyzed to determine whether the armor will provide adequate protection against trauma (without perforation/penetration of the package). The requirements of the NIJ [23] state that all depths of BFS in the support clay, obtained from fires obeying the firing requirements, must not exceed 44 mm, or if the BFS exceeds 44 mm then there must be a coefficient of 95% confidence that 80% of the depths of the imprints in the support material will be 44 mm or less. Under no circumstances should a BFS in the support material exceed 50 mm.

**Figure 17:**
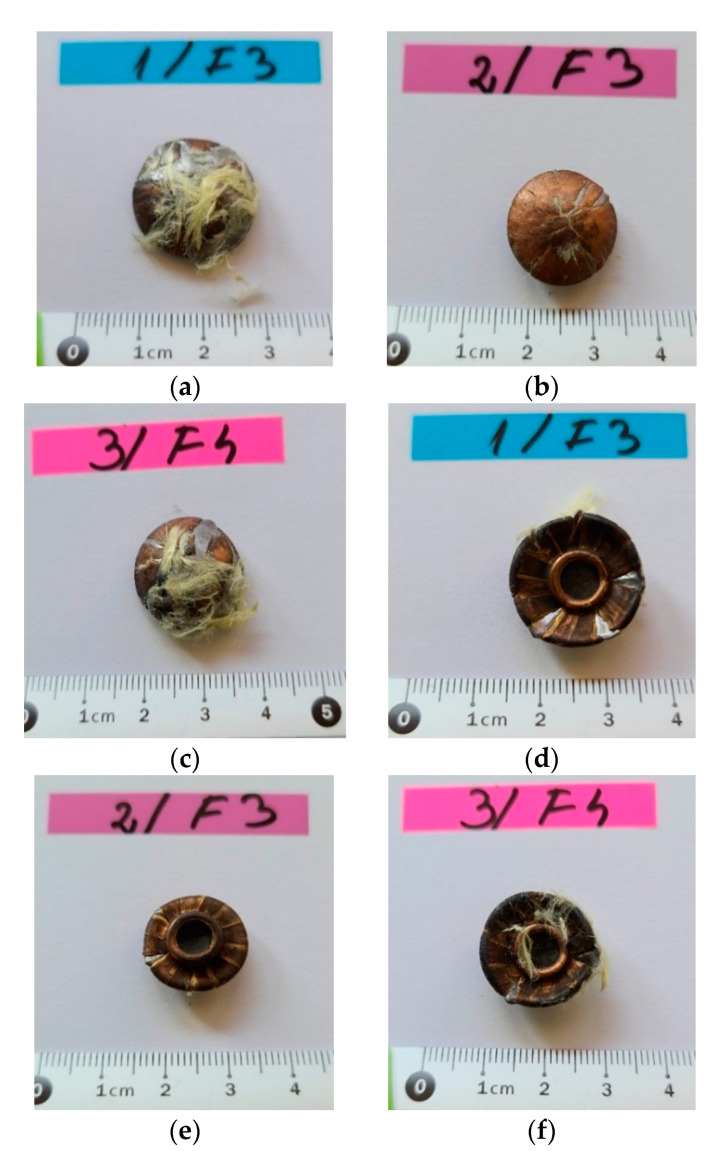
Bullets (9 mm FMJ) as recovered after testing 12-layer ballistic package: front view of the bullets (**a**–**c**); back view of the same bullets (**d**–**f**).

Following the extraction of the bullets from a package of 12 LFT SB1plus layers, the following characteristics of the recovered projectiles could be highlighted based on the analyses of the photographs in Figure 17:

- Bullets were flatted like a mushroom cap;
- Bullets had initiated fragmentation on their edges, but no fragments seemed to be separated, with the jacket and core torn off the second bullet;
- Their faces in contact with the target had trapped aramid fibers, especially from the first and second hit yarns.

The number of layers penetrated in the package influences the way the projectile is damaged, and the bullets retained within the package (Figure 17) had a very flattened shape, with peripheral ruptures of the jacket and core.

**Figure 18:**
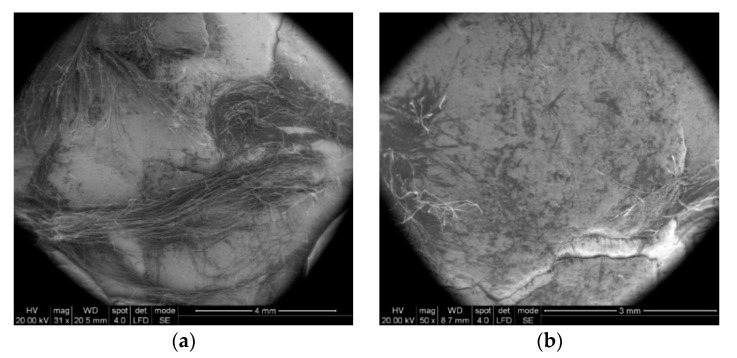
SEM details of bullet of 9 mm FMJ, as retrieved after firing. (**a**) Bullet of 2nd fire; (**b**) bullet of 3rd fire.

Figure 18 presents details of the impacted face of the projectile after testing. One may notice the embedding of fragmented aramid yarns on the projectile front. The first yarn contacting the bullet was broken near its contacting length with the bullet due to high tensile stress; this central fragment was so compressed that its fibers were forced into the projectile material and remained fixed on the bullet, even if this one was intensively deformed and cracked. The bullet from the 3rd fire kept a similar aspect of the bullet failure (local fragmentation and plastic deformation), but only several fibers were “stuck” to the bullet. Several fibers were trapped in the cracks of the projectile, both in the lead core and copper alloy jacket.

## 5. Conclusions

The main objective of this study focuses on designing and testing protection systems using advanced materials based on aramid fibers for high-impact speeds of up to 420 ± 10 m/s. The investigation of the failure mechanisms identifies current issues of protective materials, major challenges and technological problems for developing these types of systems, including the fulfilling of ballistic impact requirements according to NIJ Standard-0101.06 [23].

The purpose of the paper was to conduct an investigation on the failure processes and destructive stages of a ballistic package made of successive layers of LFT SB1plus, taking into account the particular test conditions from NIJ Standard-0101.06 [23]. The main parameter of interest was the backface signature (BFS), but also details of projectile arrest and SEM investigation could offer an argument for using this material for individual protection. For the reported tests, the maximum and minimum values for BFS were 12 mm and 24 mm, the mean value being 18.66 mm and the standard deviation being 3.8 mm. These values recommend the package for use as a protective panel for body armor for threat level IIA.

## Figures and Tables

**Table 1:** BFS of a ballistic package made of LFT SB1plus layers.

LFT SB1plus Package of 12 Layers	
Fires	BSF (mm)
1	14
2	15
3	17

## Data Availability

The data presented in this study are available on request from the corresponding author.
